# Evaluating EMG Feature and Classifier Selection for Application to Partial-Hand Prosthesis Control

**DOI:** 10.3389/fnbot.2016.00015

**Published:** 2016-10-19

**Authors:** Adenike A. Adewuyi, Levi J. Hargrove, Todd A. Kuiken

**Affiliations:** ^1^Department of Biomedical Engineering, Northwestern University, Chicago, IL, USA; ^2^Center for Bionic Medicine, Rehabilitation Institute of Chicago, Chicago, IL, USA; ^3^Feinberg School of Medicine, Northwestern University, Chicago, IL, USA; ^4^Department of Physical Medicine and Rehabilitation, Northwestern University, Chicago, IL, USA

**Keywords:** pattern recognition, electromyography, partial-hand amputee, myoelectric control, intrinsic hand muscles, feature selection

## Abstract

Pattern recognition-based myoelectric control of upper-limb prostheses has the potential to restore control of multiple degrees of freedom. Though this control method has been extensively studied in individuals with higher-level amputations, few studies have investigated its effectiveness for individuals with partial-hand amputations. Most partial-hand amputees retain a functional wrist and the ability of pattern recognition-based methods to correctly classify hand motions from different wrist positions is not well studied. In this study, focusing on partial-hand amputees, we evaluate (1) the performance of non-linear and linear pattern recognition algorithms and (2) the performance of optimal EMG feature subsets for classification of four hand motion classes in different wrist positions for 16 non-amputees and 4 amputees. Our results show that linear discriminant analysis and linear and non-linear artificial neural networks perform significantly better than the quadratic discriminant analysis for both non-amputees and partial-hand amputees. For amputees, including information from multiple wrist positions significantly decreased error (*p* < 0.001) but no further significant decrease in error occurred when more than 4, 2, or 3 positions were included for the extrinsic (*p* = 0.07), intrinsic (*p* = 0.06), or combined extrinsic and intrinsic muscle EMG (*p* = 0.08), respectively. Finally, we found that a feature set determined by selecting optimal features from each channel outperformed the commonly used time domain (*p* < 0.001) and time domain/autoregressive feature sets (*p* < 0.01). This method can be used as a screening filter to select the features from each channel that provide the best classification of hand postures across different wrist positions.

## Introduction

Pattern recognition-based myoelectric control of externally powered prostheses has demonstrated remarkable potential to restore function to individuals with upper-limb amputations. This control method has shown promise in laboratory settings (Kuiken et al., 2009; Scheme and Englehart, 2011), and a pattern recognition myoelectric controller is now clinically available for individuals with high-level upper-limb amputations (Uellendahl et al., 2016). However, this population comprises less than 10% of all upper-limb amputations in the United States (Dillingham et al., 2002; Ziegler-Graham et al., 2008). The majority of amputations are distal to the wrist (i.e., partial-hand amputations) (Dillingham et al., 2002). Since this level of amputation can involve a variety of clinical presentations, it is difficult to treat successfully with a prosthesis (Lake, 2004). Though, partial-hand amputations are often termed “minor” amputations (Ziegler-Graham et al., 2008), successful treatment is of significant importance because the effects of partial-hand amputation on employment and self-image are comparable to those of more proximal amputations (Burger et al., 2007; Hebert and Burger, 2016). Partial-hand amputees perceive themselves to be at a higher disability level than do individuals with unilateral transradial or transhumeral amputations (Davidson, 2004; McFarland et al., 2010), they are more likely to reject their prosthesis (Biddiss and Chau, 2007), and more than half are unable to return to their previous occupation (Burger et al., 2007).

Though externally powered myoelectric prostheses for more proximal upper-limb amputees have been commercially available for decades (Parker and Scott, 1986), they have only recently become available to partial-hand amputees, in part because of the technological complexities of replacing the motor function of a finger within the size limits of a prosthetic digit (Uellendahl and Uellendahl, 2012). Externally powered partial-hand prostheses, such as the i-limb quantum (Touch Bionics Inc.) and Vincentpartial (Vincent Systems GmbH) have independently functioning digits and, thus, offer a wide range of articulated grasps not previously available to partial-hand amputees. Commercial prostheses use conventional control algorithms that use an estimate of the EMG amplitude for proportional control of the speed of an actuated joint (Phillips et al., 2012; Uellendahl and Uellendahl, 2012). Though pattern recognition control has the potential to intuitively restore control of more degrees of freedom than conventional methods (Englehart and Hudgins, 2003; Hargrove et al., 2007; Kuiken et al., 2009), it has not yet been shown to be sufficiently robust for partial-hand prosthesis control.

Partial-hand amputees often retain the ability to move their wrists, and preservation of residual wrist motion is critical for functional everyday activities. Montagnani et al. (2015) showed that when non-amputees are limited to two degrees of freedom at the wrist (pronation/supination and flexion/extension) and one degree of freedom at the hand (open/close), they perform similarly to when they are limited to a one degree-of-freedom wrist (rotation) coupled with their intact, twenty-two degree-of-freedom hand. Thus, a clinically successful partial-hand pattern recognition control system must maintain high performance while allowing the individual to use their wrist. Our previous studies demonstrate that varying wrist position adversely affects pattern recognition performance in offline and real-time virtual studies, though the severity of this wrist position effect is diminished by training the classifier with data from multiple wrist positions and combining EMG data from the extrinsic and intrinsic muscles of the hand (Adewuyi et al., 2016; Earley et al., 2016).

The selection of effective features and robust classifiers are critical in the design of pattern recognition-based control systems. Previous studies that investigated classifiers, such as artificial neural networks (Hudgins et al., 1993), hidden Markov models (Chan and Englehart, 2005), linear discriminant analysis (LDA) (Englehart and Hudgins, 2003), support vector machines (Al-Timemy et al., 2013), Gaussian mixture models (Huang et al., 2005), and quadratic discriminant analysis (Scheme and Englehart, 2011) found little difference in classification error between different classifiers within non-amputee and amputee groups (Scheme and Englehart, 2011). An LDA classifier is used most commonly because it provides a good balance between classification performance and computational efficiency. However, because most studies have focused on individuals with more proximal amputations, it remains unclear whether these findings are true for partial-hand amputees whose forearm muscle activity is significantly modulated by wrist movement during a task (Mogk and Keir, 2003; Johnston et al., 2010).

Pattern recognition of EMG signals is dependent on the user’s ability to generate repeatable and differentiable muscle contractions. Effective EMG features are those that both provide unique information about limb motion and are minimally sensitive to factors that degrade performance by altering the EMG signals – such as electrode shift (Young et al., 2012), muscle fatigue, muscle contraction effort (Tkach et al., 2010), force variation (Al-Timemy et al., 2015), and limb position (Al-Angari et al., 2016). The robustness of numerous features to such factors has been evaluated; however, typically, the performance of features and feature combinations are evaluated across all channels. Few studies have investigated the importance of selecting individual features from different channels, and no studies, to our knowledge, have specifically evaluated which feature subsets are most robust to changes in wrist position. To search for important subsets in the feature/channel space, Oskoei et al. (2013) used separability indices and classification rate as objective functions and a genetic algorithm as a search strategy, whereas Khushaba and Al-Jumaily (2007) used classification rate as an objective function and particle swarm optimization as an evolutionary computation search technique. Both of these studies aimed to increase the efficiency of pattern recognition by finding optimal feature subsets, but the selection of best features and channels was not done simultaneously. More recently, Al-Angari et al. (2016) used feature/channel subset selection (using correlation-based and distance-based methods) to determine whether selecting optimal features from each channel would improve the limb position effect.

This work evaluates several strategies in non-amputees and partial-hand amputees for improving classification of hand grasps performed with varying wrist positions. In this study, we (1) compare the performance of linear and non-linear classification techniques and (2) evaluate the performance of optimal EMG feature subsets that are most robust to wrist position variation.

## Materials and Methods

### Data Collection

Data from non-amputee subjects, previously collected by Adewuyi et al. (*n* = 7) (Adewuyi et al., 2013, 2016) and Earley et al. (*n* = 9) (Earley et al., 2014) using similar protocols, were combined and used for this study. According to Adewuyi et al., nine self-adhesive bipolar surface Ag/AgCl EMG electrodes (Bio-Medical Instruments) were evenly spaced around the dominant forearm with an inter-electrode distance of 2.5 cm: five electrodes on the proximal forearm, 2–3 cm distal to the elbow and four electrodes on the distal forearm, 7–8 cm proximal to the wrist. However, for the data from Earley et al., eight self-adhesive bipolar surface Ag/AgCl EMG electrodes (Bio-Medical Instruments) were evenly spaced around the forearm: six electrodes on the proximal forearm and two electrodes on the distal forearm (one on the anterior side and one on the posterior side). EMG data from intrinsic hand muscles were recorded with four electrode pairs on the hand. Two electrode pairs were placed on the palmar side (over the thenar and hypothenar eminence) and two electrode pairs were placed on and dorsal sides (over the first and third dorsal interossei). Data from partial-hand amputee subjects (*n* = 4), previously obtained by Adewuyi et al., were also evaluated (Adewuyi et al., 2013, 2016). All subjects gave written consent, and experiments were performed at the Rehabilitation Institute of Chicago under an approved Northwestern University Institutional Review Board (IRB) protocol.

### EMG Signal Processing

EMG signals were acquired using a custom-built EMG amplifier with a software gain of 2000× for each channel. All EMG data were digitally sampled at 1000 Hz using a custom-built A/D converter based on a TI AD1298 24-bit bioamplifier chip and band pass filtered (30–350 Hz) with a Type 1, eighth-order Chebyshev filter.

### Procedure

Custom-designed computer software was used to visually prompt subjects to perform two functional hand grasps (key grip and chuck grip), one open hand posture, or a rest posture. All four hand postures were performed with a neutral wrist position and repeated while the subjects held their wrist in the following comfortable positions: flexion, extension, pronation, supination, abduction, and adduction, for a total of seven wrist positions. Each hand posture was held for 3 s. Subjects from Earley et al. performed four repetitions of each hand posture in each wrist position (Earley et al., 2014), and subjects from Adewuyi et al. performed 10 repetitions of each hand posture in each wrist position (Adewuyi et al., 2013, 2016).

### Data Analysis

Offline analyses were performed using MATLAB 2015a software (The Mathworks, Natick, MA, USA). For all conditions, data were segmented into 200-ms windows with a 20-ms frame increment (Smith et al., 2011).

#### Effect of Classifier Type on Classification Error

A combination of four EMG time domain (TD) features [mean absolute value (MAV), number of zero crossings, waveform length (WL), and number of slope-sign changes] and six coefficients of a sixth-order autoregressive (AR) model features (hereafter called TDAR features) was extracted from each EMG data window. Four classifiers were examined: (1) an LDA classifier, (2) a quadratic discriminant analysis classifier (QDA), (3) a multilayer perceptron neural network with linear activation functions in its one hidden layer (LNN), and (4) a multilayer perceptron artificial neural network with non-linear hyperbolic tangent sigmoid activation functions in its one hidden layer (MLPANN). The LDA was selected because it is the most commonly used for the classification of limb movements using EMG. It was compared to a QDA because they make very similar assumptions about the data except that it allows non-linear boundaries between data. These were compared to a LNN and MLPANN because they are on the opposite side of the spectrum in that they make no assumptions about the underlying distribution of the data.

All classifiers were trained using data from (1) only extrinsic muscle EMG data, (2) only intrinsic muscle EMG data, or (3) a combination of all extrinsic and intrinsic muscle EMG data. Data were divided into training data sets (50% of all data), testing data sets (30% of all data) and validation data sets (20% of all data). The validation data sets were used to minimize overfitting of the neural networks; training of the neural networks stopped once the classification error of the validation sets began to increase. First, the training and testing data sets were used to train and test the classifiers, respectively. The other 50% of the data (previously used for testing and validation in the first group) was used for training and 30% of the data (previously part of the training set in the first group) was used for testing. The results of these two groups were then averaged. Seven hidden layer neurons were empirically chosen for the MLPANN, and the LNN had four neurons in its hidden layer. Since the LNN has linear activation functions, it simply maps the weighted inputs to the output of each neuron and is, thus, mathematically equivalent to a reduced two-layer input–output model (Haykin, 1999). The neural networks were trained using scaled conjugate gradient descent (Møller, 1993).

An exhaustive search was performed to determine the optimal number of wrist positions needed for classifier training. An LDA classifier was trained using data from one to seven wrist positions and tested on data from all seven wrist positions. All possible combinations of data from *n* wrist positions were evaluated, and the combination with the lowest error was chosen for each subject and plotted as a function of number of wrist positions.

#### Effect of EMG Feature Subset on Classification Error

Twenty five time and frequency domain features were extracted from each EMG channel. Nineteen of these features were: MAV, zero crossings (ZC), slope-sign changes (SSC), WL, Willison amplitude (WAMP), root-mean-square (RMS), variance (VAR), v-order (order of 3), log-detector (LogDet), AR coefficients (order of 6), mean frequency (MnF), median frequency (MdF), peak frequency (PF), and mean power (MP). The frequency domain features MnF, MdF, PF, and MP were derived from the short-time Fourier transform using Hamming windows. Previous studies have shown that feature sets based on the short-time Fourier transform perform better than TD features and are comparable to feature sets based upon the wavelet transform and the wavelet packet transform (Englehart et al., 1999). The remaining six features were a set of power spectrum descriptors (PSD) proposed by Al-Timemy et al. (2015). These features were derived as the orientation between features extracted from a non-linearly mapped EMG record and the original EMG record and as such the resultant features were shown to be less affected by different contraction efforts.

Two main approaches can be used to select an optimal feature subset: the filter or the wrapper. The filter approach typically evaluates features based on their discriminative power using their content (e.g., within- and between-cluster separability, distance measures). The wrapper approach applies a classifier to evaluate feature subsets by minimizing classification error. Here, we used the Bhattacharyya distance as a filter function and an LDA as a wrapper function.

The Bhattacharyya distance is used as an important measure of the separability between distributions (Bhattacharyya, 1946; Park and Lee, 1998). Because it evaluates features based on their discriminative power using their content, it is independent of the classifier type and can be generalized to other classifiers. We evaluated and defined the separability index for each feature/channel combination (SI) as:

SI=minX=1:Nc−1,Y=X+1:Nc DB{cX,cY}

where *N*_c_ is the total number of classes available, which for this study was 4. D_B_{*c*_1_,*c*_2_} is the Bhattacharyya distance between the distributions of classes *c_X_* and *c_Y_*. *SI* is, therefore, the minimum separability between all classes, for a given feature/channel combination. This was calculated using data from all the wrist positions. The larger the separability index, the greater the feature’s ability to distinguish one class from another, thereby leading to an increased likelihood of correct class selection by a pattern recognition classifier. The separability indices were sorted in descending order. The final number of feature/channel combinations selected from this ordered list was equivalent to the number of features in the TDAR feature sets.

The wrapper method used an LDA classifier in combination with a feature selection algorithm based on the sequential forward searching (SFS) method (John et al., 1994). In SFS method, there are two sets: set A that is initially empty and set B that includes all the features. This algorithm employs an iterative search method where it selects the feature from set B that produces the minimum classification error as the first selected feature in set A. It then pairs each of the remaining features in set B with all the features in set A. The feature in set B paired with all the features in set A that generates the minimum classification error is identified and moved to set A. In each iteration, one feature in set B is selected and added to set A as the most informative feature. This method, thus, does not just select individual features that have the lowest classification error but selects features that result in the lowest classification error when paired with other features. This was performed using EMG data from the (1) extrinsic, (2) intrinsic and, (3) combination of the extrinsic and intrinsic muscles. In total, five feature sets were compared. They were as follows: TDAR features, TD features (MAV, ZC, SSC, and WL), SI features (features selected from each channel based on separability index), SFS features (features selected from each channel using the SFS method), and all features. The final number of features in the SI and SFS feature subsets was equivalent to the number of features in the TDAR feature sets. The five feature subsets were compared using an LDA classifier alone.

To test the reliability of these feature sets, sensitivity and specificity were calculated where sensitivity was defined as the number of recognized true hand motion classes divided by the total number of true hand motion classes. Specificity was defined as the number of rejected false hand motion classes divided by the total number of false hand motion classes.

To determine which features were most important, the features were added one-by-one as inputs into an LDA classifier in the order of their separability index or in the order of selection by the SFS method. Principal component analysis (PCA) was also used to transform the data into a new coordinate system such that the greatest variance in the data was explained by the first coordinate and the least variance in the data was explained by the last coordinate. The newly transformed coordinates were added one-by-one, as feature inputs into an LDA classifier in descending order of the amount of variance explained by each principal component. The minimum classification error was determined for all methods and the feature set was reduced to the set of X features that decreased error by 99%. This was done separately for extrinsic, intrinsic, and combination extrinsic and intrinsic muscle EMG data for non-amputees and amputees. The frequency of selection of each feature in this set of X features was determined and averaged across subjects.

#### Effect of EMG Feature Subset on Classification Error

To determine the effect of classifier type on classification error, a two-way repeated measures analysis of variance (ANOVA) test was performed with subject as a random effect, and muscle set and classifier type as fixed effects. This analysis was performed separately for amputees and non-amputees. To determine the effect of feature set on classification error, a two-way repeated measures ANOVA test was performed with subject as a random effect, and muscle set and feature set as fixed effects. *Post hoc* comparisons were made using a Bonferroni correction factor to determine significance. All analyses were performed separately for amputees and non-amputees using Minitab 17.3.1 (Minitab Inc. PA, USA), with a significance level set at α = 0.05.

## Results

### Effect of Classifier Type, Muscle Set, and Wrist Position on Classification Accuracy

**Figure 1:**
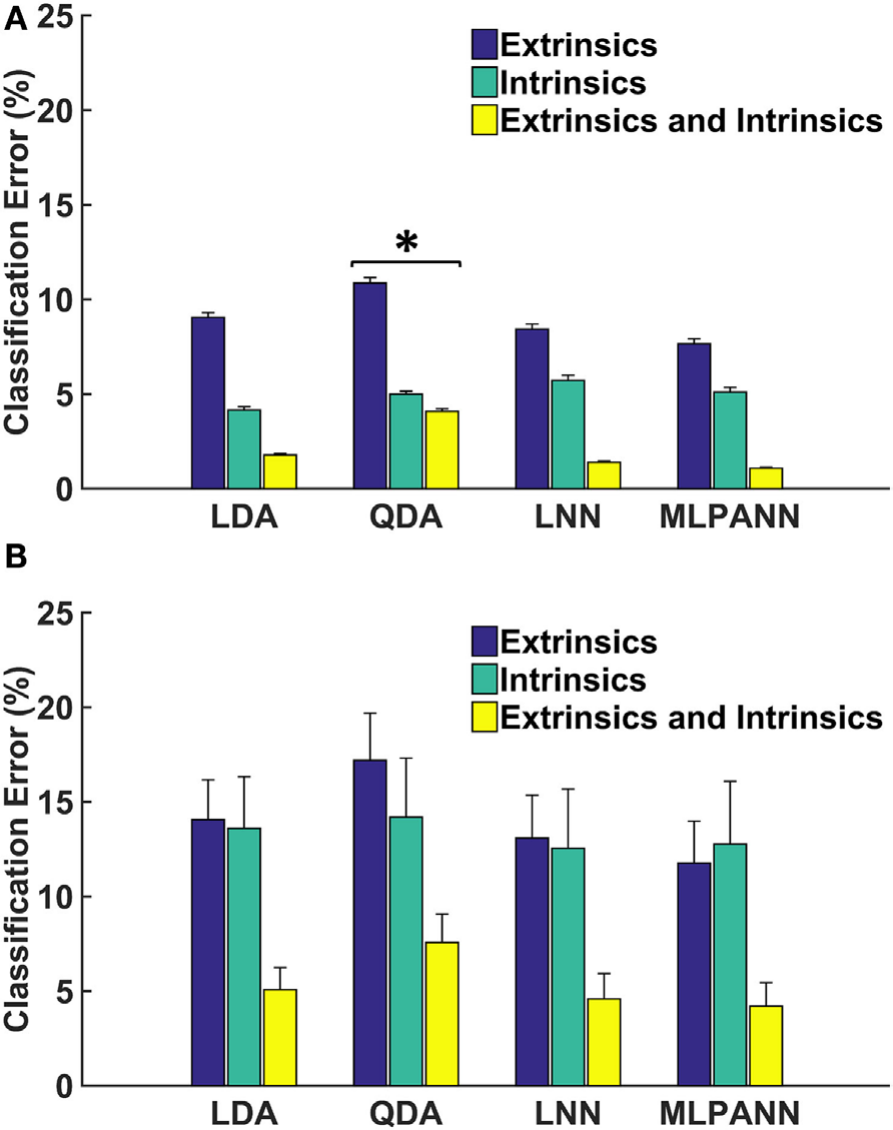
**Linear and non-linear offline classification of four hand postures**. **(A)** and **(B)** show results from 16 non-amputees and 4 partial-hand amputees (including 1 bilateral partial-hand amputee), respectively. Each classifier was trained and tested using data from seven wrist positions. LDA, linear discriminant analysis; QDA, quadratic discriminant analysis; LNN, neural network with linear activation functions; MLPANN, neural network with non-linear activation functions. Error bars represent SE (*significantly lower than LDA, LNN, and MLPANN).

For non-amputees, performance was comparable across classifiers, except that the QDA performed significantly worse than all other classifiers. The combination of extrinsic and intrinsic muscle EMG performed significantly better than either intrinsic or extrinsic muscle EMG alone (*p* < 0.001). Using EMG from intrinsic muscles alone was significantly better than EMG from extrinsic muscles alone (Figure 1A) (*p* < 0.001). There was no significant interaction between the two factors (*p* = 0.06). For amputee subjects, the QDA also performed worse than all other classifiers, though this was not statistically significant (*p* = 0.2). Performance using combined EMG data from extrinsic and intrinsic muscles was significantly better than using intrinsic or extrinsic muscle EMG alone. Unlike the non-amputee data, there was no difference in performance when using EMG from extrinsic or intrinsic muscles (*p* = 0.86) (Figure 1B).

**Figure 2:**
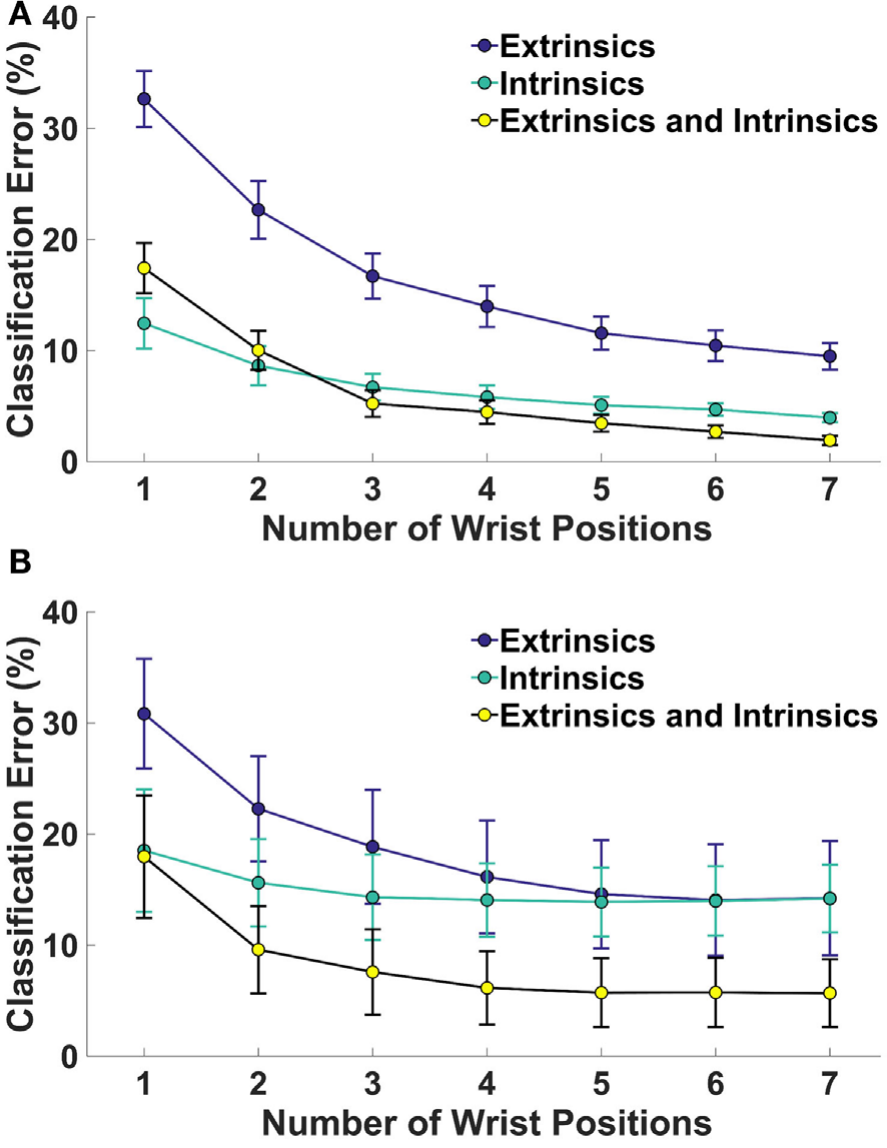
**Classification error for 4 hand grasp classes as a function of number of wrist positions for (A) 16 non-amputees and (B) 4 partial-hand subjects**. Error bars represent SE.

Figure 2 shows the relationship between the number of wrist positions and classification error. For amputees, classification error decreased as the number or wrist positions increased, but no significant decrease in error occurred when more than four, two, or three positions are included for extrinsic (*p* = 0.07), intrinsic (*p* = 0.06), and the combination of extrinsic and intrinsic muscle EMG (*p* = 0.08), respectively. For non-amputees, error continued to significantly decrease with each additional wrist position for the extrinsic muscle and combined extrinsic and intrinsic muscle EMG. For the intrinsic muscles, no significant decrease in error occurred when more than four wrist positions were included (*p* = 0.09).

### Effect of Feature Selection on Classification Error

**Figure 3:**
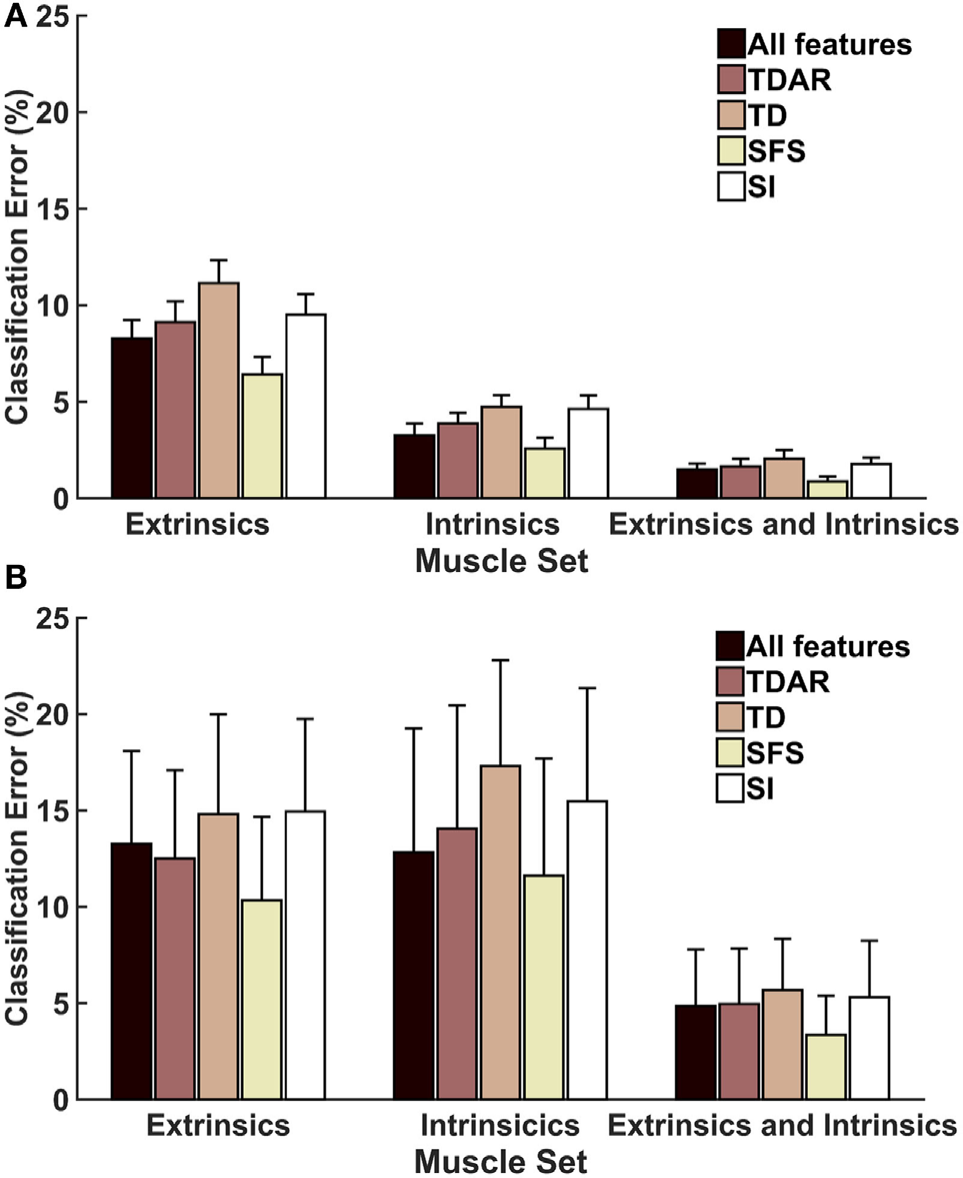
**Average classification error for (A) 16 non-amputees and (B) 4 partial-hand amputees for 5 feature sets**. LDA classifiers were trained and tested with data from seven wrist positions. TDAR, time domain and autoregressive features; TD, time domain features; SFS, optimal feature/channel combinations as determined by sequential forward search algorithm; SI, optimal feature/channel combinations as determined by the separability index. Error bars represent SE.

Figure 3 shows the average classification errors across five EMG feature sets. For both amputees and non-amputees, there was a main effect of muscle set and feature set and no significant interaction between these factors (*p* = 0.98, *p* = 0.1, respectively). The SFS feature set performed better than all other features, including feature sets that used all features, and performed significantly better than the TDAR feature set, TD feature set, and SI feature set (Table 1). For amputees, the SFS feature set also performed the best but was only significantly better than the TD feature set (*p* = 0.03). The analysis of the sensitivity and specificity of the feature sets revealed the same trends observed with classification accuracy and are presented in Table S1 in Supplementary Material.

**Table 1:** ***P*-value table for pair-wise comparisons between different EMG feature sets for non-amputees**.

	All features	TDAR	TD	SFS	SI
All features	–	0.7	**<0.001**	0.12	0.4
TDAR		–	0.9	**<0.01**	0.94
TD			–	**<0.001**	0.65
SFS				–	**<0.001**
SI					–

*Bold values in the table are statistically significant (i.e. p < 0.05)*.

**Figure 4:**
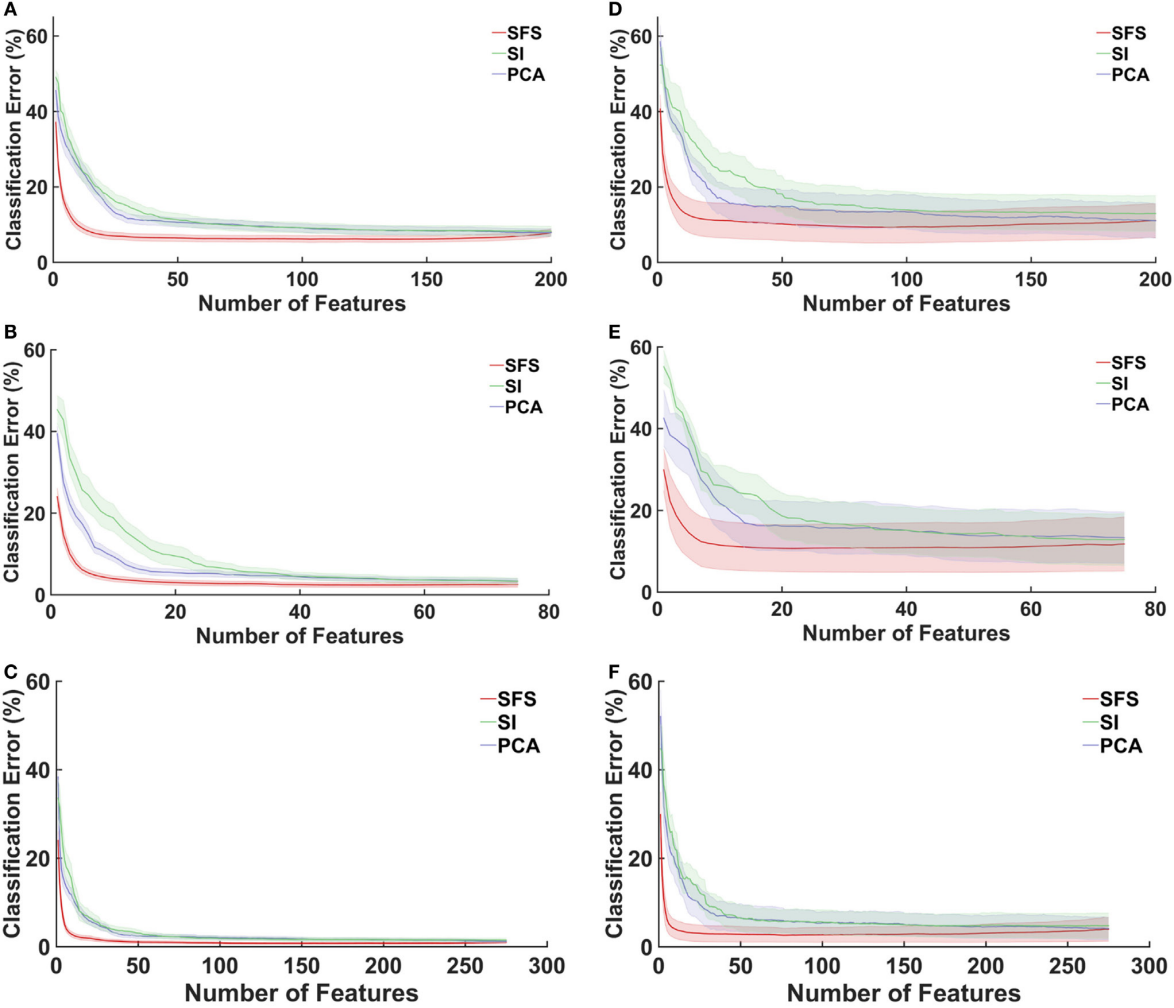
**Average classification errors as a function of number of feature numbers for three feature selection methods**. SFS, feature selection using sequential forward selection; SI, feature selection using the separability indices of each feature; PCA, principal component analysis. LDA classifiers were trained and tested with data from seven wrist positions to recognize four motion classes. Shaded error bars represent SE. **(A)** Extrinsics: non-amputees, **(B)** intrinsics: non-amputees, **(C)** extrinsics and intrinsics: non-amputees, **(D)** extrinsics: amputees, **(E)** intrinsics: amputees, and **(F)** extrinsics and intrinsics: amputees.

Figure 4 shows the relationship between classification error and number of features using SFS, separability indices (SI), and PCA as feature selection methods. For both non-amputees and amputees, and across all muscle groups, feature selection using SFS reached a minimum error rate at a much faster rate and with fewer features than the PCA or SI methods. For example, with the SFS method, a minimum error of 6.18% was achieved with 139 features, but with only 36 features, classification error had decreased by 99%, to 6.625%.

**Figure 5:**
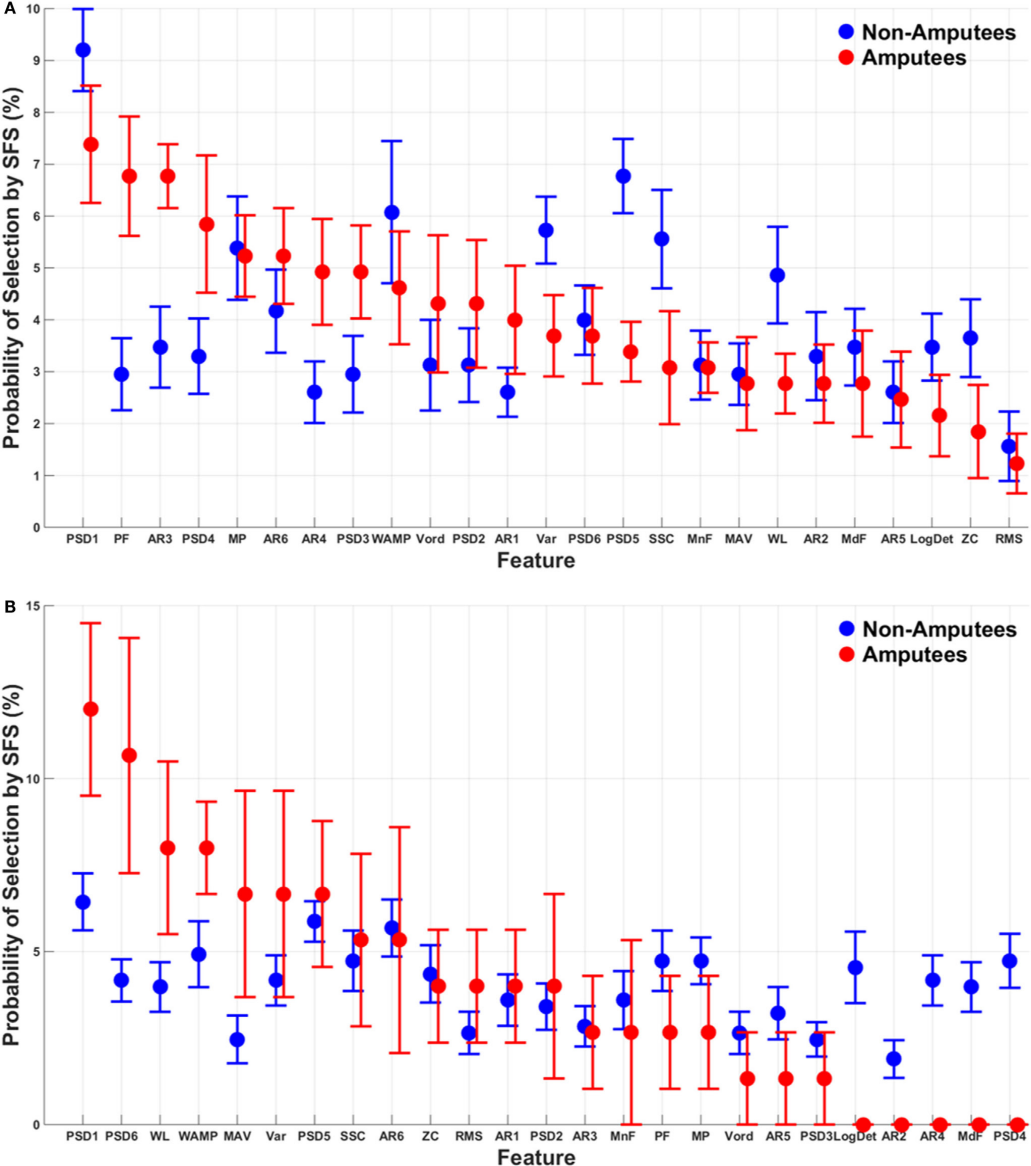

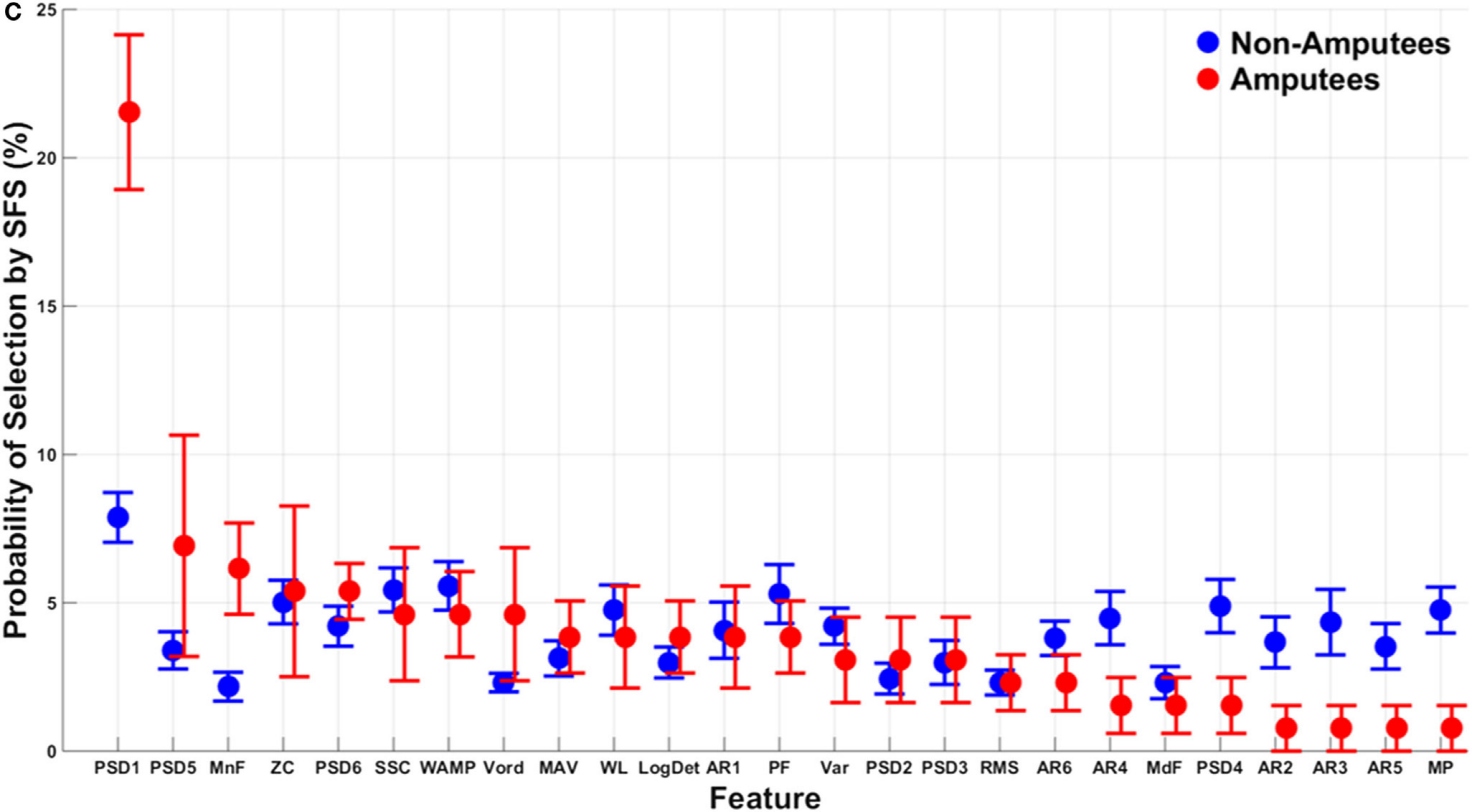
**Probability of selection of the 25 features using the SFS method**. Features are ordered from most to least often selected for amputee and non-amputee subjects. Mean absolute value (MAV), zero crossings (ZC), slope-sign changes (SSC), waveform length (WL), Willison amplitude (WAMP), root-mean-square (RMS), variance (VAR), v-order (V-ord, order of 3), log-detector (LogDet), autoregressive (AR1–AR6) coefficients, mean frequency (MnF), median frequency (MdF), peak frequency (PF), mean power (MP), and power spectrum descriptors (PSD1–PSD6). Error bars represent SE. **(A)** Extrinsics, **(B)** intrinsics, and **(C)** extrinsics and intrinsics.

The probability of selection of each of the 25 features in the subset of features that account for 99% of the maximum classification accuracy, averaged across subjects, is presented using the SI method (Figure S1 in Supplementary Material) and using the SFS method (Figure 5). Using the SI method, the features that were most and least often selected were generally consistent between amputees and non-amputees. The autoregressive features were much less likely to be selected for both non-amputees and amputees using the SI method than using the SFS method. Moreover, though the importance of the features was relatively consistent across muscles using the SI method, the importance of features differed drastically across muscle sets for the SFS method for amputees. For example, the MAV, WL, and SSC features were, respectively, the 18th, 19th, and 16th most often chosen feature from extrinsic muscle EMG data for amputees, but were the 5th, 3rd, and 8th most often chosen features, respectively, from intrinsic muscle EMG data in amputees. Some features, however, were consistently selected across EMG datasets, such as the first power spectrum descriptor (PDS1), which was the most commonly selected feature across all muscle sets, non-amputees and amputees.

## Discussion

The application of pattern recognition techniques for control of externally powered myoelectric partial-hand prostheses promises to restore more function to partial-hand amputees than previously available. This work evaluated two approaches for improving the robustness of pattern recognition control against the effect of wrist position: (1) comparison of linear and non-linear classification schemes and (2) the selection of the best features taken from each channel.

Overall, the performance of all classifier types was comparable for amputees and non-amputees though the QDA performed worse than all other classifiers. This may be because unlike the LDA, the QDA is a more complex model that allows for the heterogeneity of covariance matrices for each class of data. Consequently, it requires more data to estimate more parameters and achieve high accuracies. It is also possible that the QDA performed worse because of overfitting of the training data. Although the average performance of non-amputees and amputees was different, the relative performance of different classifiers was consistent within the two groups. These findings are consistent with those of Scheme and Englehart (2011), who evaluated offline classifier performance for individuals with transradial amputations.

Among numerous possible combinations of features, TD and TDAR features (MAV, SSC, ZC, WL, and autoregressive coefficients) are commonly used. Our results show that the optimal feature set determined by sequentially adding one feature from each channel using the SFS method outperformed all other feature sets. Few studies have investigated the importance of selecting the best features from different channels. Al-Angari et al. (2016) used the Mahalanobis distance and a correlation-based method to determine the best features in each channel that were most resistant to changes in limb position. They also found a significant variation in the probability of selection of the AR features using the two feature selection methods. This is most likely because the Bhattacharyya distance, such as the Mahalanobis distance, looks at the separability of different classes for each feature, whereas SFS indirectly considers the mutual information between each feature and class and selects the feature that best improves error in conjunction with other features already in the chosen set.

Not only are some features more important only in the context of other features, but also the muscle group from which EMG is extracted greatly affects feature selection. The commonly used time-domain features MAV, WL, ZC, and SSC, which have been found to be effective in classifying hand postures, were among the least important features selected from the extrinsic muscle EMG, and the most important features selected from the intrinsic muscle data. This is most likely because these features are significantly affected by changes in extrinsic muscle EMG in different wrist positions, but the intrinsic muscles, which do not cross the wrist joint, are less affected by changes in wrist position. Because the majority of partial-hand amputations are caused by trauma (Ziegler-Graham et al., 2008), the intrinsic muscles can be severely damaged, or absent and, thus, not viable for EMG-based control. In such cases, it becomes more important to optimize control using extrinsic muscle EMG by selecting the appropriate features.

An optimal feature is one that both allows for discrimination between hand postures across multiple wrist positions as well as providing information that is distinct from other features. Methods, such as the SFS method, that select the best performing features that provide distinct discriminatory information about hand grasps patterns could be useful for proper pre-selection of features for classification of different hand postures in different wrist positions. We found that the time-dependent PSDs proposed by Al-Timemy et al. (2015) were reasonably well selected for both SI and SFS methods across all muscle groups, suggesting that they are less affected by changes in wrist position and provide good classification of hand grasps. The set of PSDs are extracted directly from the TD using Fourier transform relations and Parseval’s theorem and, thus, keep computational costs low. Given their consistently good performance across muscle sets and subject groups, these features should be taken into consideration for future clinical implementation of pattern recognition-based systems for partial-hand prostheses.

We collected data from seven wrist positions, which can be burdensome for the user especially as the user trains the pattern recognition system with more hand grasps. We found that for amputee subjects, training in more than two to four positions provided no significant additional improvement. This study has a potential limitation in that the analyses for non-amputees were performed offline. Some previous research has demonstrated a minimal correlation between offline performance and usability with a virtual task (Lock et al., 2005; Jiang et al., 2014); however, other studies have shown significant correlation between offline classification error and real-time control (Smith et al., 2011; Young et al., 2011). The real-time implementation would involve the pre-selection of appropriate features from each channel using SFS. Once complete, real-time classification would proceed only using those preselected features. As this method only selects relevant features, it would involve the selection of a fewer number of features than TDAR features from all channels. Given the improvement in offline performance using the SFS method particularly for the extrinsic muscles, we would expect that preselecting features that are least sensitive to wrist position would result in better performance than the TDAR features though the relationship between offline and real-time performance is unclear. Thus, further analysis of data from amputees completing tasks with the wrist in different positions in a virtual environment or with a physical prosthesis is warranted.

## Conclusion

In order for pattern recognition techniques to be used for control of partial-hand prostheses, the control system must be robust enough to main good control when the user moves their wrist. This research study compared the performance of linear and non-linear classification schemes and evaluated the performance of different EMG feature sets for improving pattern recognition control of hand grasps in multiple wrist positions. We found that the commonly used LDA classifier performed just as well as linear and non-linear artificial neural networks for amputees and non-amputees. We also found that selecting the best features from each channel using an SFS algorithm resulted in significant improvements over the commonly used TD feature sets and optimal feature sets. Finally, our results suggest that some of the widely used TD features are better suited for use with intrinsic muscle EMG data than extrinsic muscle data for good control across multiple wrist positions.

## Author Contributions

AA helped in conceiving the study concept, collecting, analyzing, and interpreting the data; drafting the manuscript; and obtaining funding. LH helped in conceiving the study concept, interpreting the data, critically revising the manuscript for important intellectual content, obtaining funding, and supervising the study. TK helped in conceiving the study concept, interpreting the data, obtaining funding, and supervising the study. All authors read and approved the final manuscript.

## Conflict of Interest Statement

LH and TK have ownership interest in Coapt LLC., a start-up company that sells myoelectric pattern recognition control systems. No Coapt products were used as part of this research. The remaining author declares that the research was conducted in the absence of any commercial or financial relationships that could be construed as a potential conflict of interest. The handling editor declared a past co-authorship with the author TK and states that the process nevertheless met the standards of a fair and objective review.

## Supplementary Material

Table S1 **Average specificity and sensitivity of the five feature sets for all hand motion classes, averaged across subjects**.

Figure S1 **Probability of selection of the 25 features using the separability indices (SI)**. Features are ordered from most to least often selected for amputee subjects. Mean absolute value (MAV), zero crossings (ZC), slope-sign changes (SSC), waveform length (WL), Willison amplitude (WAMP), root-mean-square (RMS), variance (VAR), v-order (V-ord, order of 3), log-detector (LogDet), autoregressive (AR1–AR6) coefficients, mean frequency (MnF), median frequency (MdF), peak frequency (PF), mean power (MP), and power spectrum descriptors (PSD1–PSD6). Error bars represent SE. **(A)** Extrinsic muscles, **(B)** intrinsic muscles, and **(C)** extrinsic and intrinsic muscles.

## Abbreviations

ANOVA, analysis of variance; AR, autoregressive; EMG, electromyogram; LDA, linear discriminant analysis; LNN, linear neural network; LogDet, log-detector; MAV, mean absolute value; MdF, median frequency; MnF, mean frequency; MP, mean power; PF, peak frequency; PSD, power spectrum descriptors; QDA, quadratic discriminant analysis; RMS, root-mean-square; SFS, sequential forward searching; SI, separability index; SSC, slope-sign changes; TD, time domain; TDAR, time domain and autoregressive; VAR, variance; V-ord, V-order; WAMP, Willison amplitude; WL, waveform length; ZC, zero crossings.
