# Supplementary figures and images for: Evaluating EMG Feature and Classifier Selection for Application to Partial-Hand Prosthesis Control
Source: Front Neurorobot. 2016 Oct 19;10:15. doi: 10.3389/fnbot.2016.00015 (PMC5069722; doi:10.3389/fnbot.2016.00015)

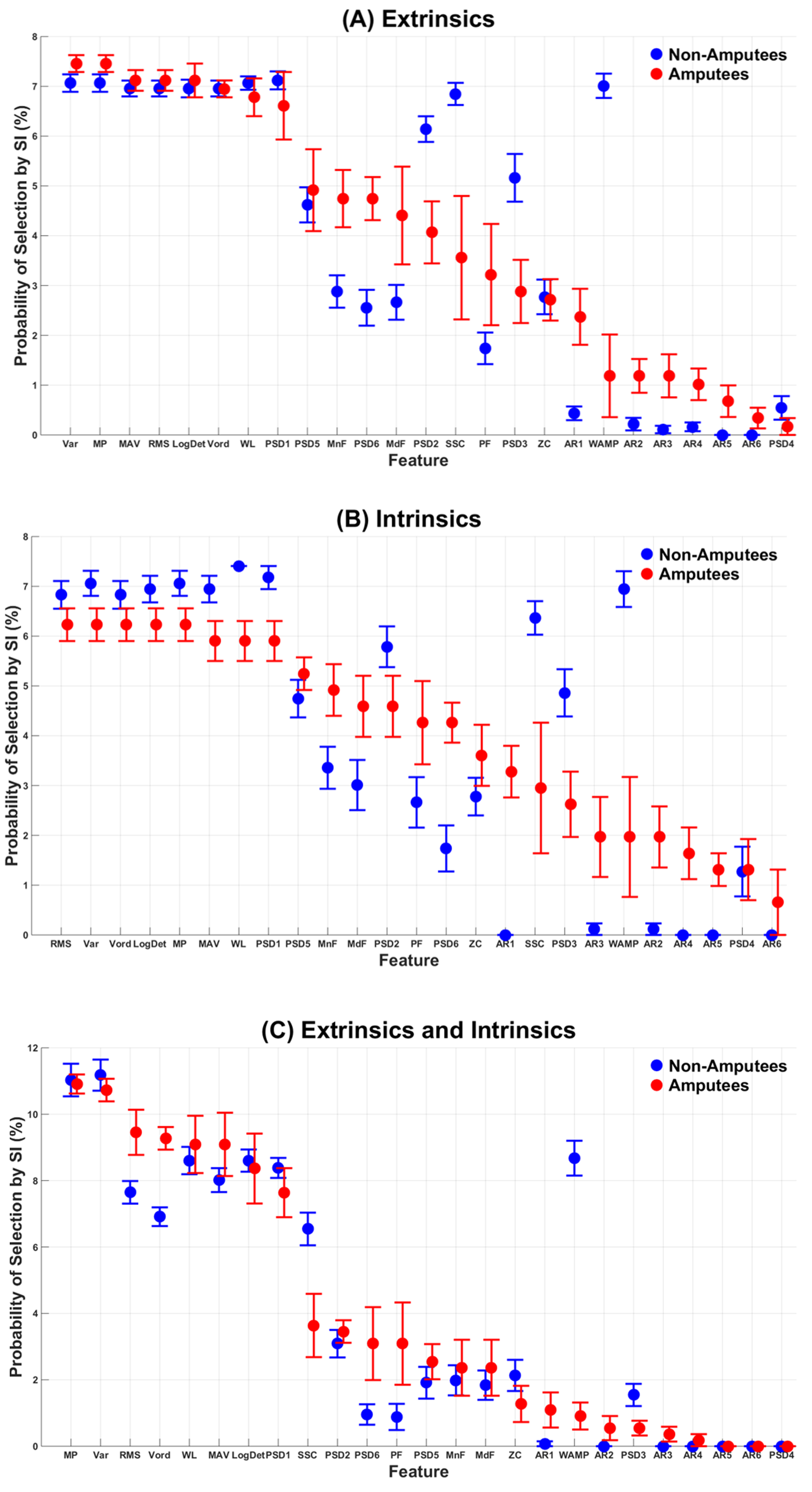

Supplement: Figure S1 — Probability of selection of the 25 features using the separability indices (SI). Features are ordered from most to least often selected for amputee subjects. Mean absolute value (MAV), zero crossings (ZC), slope-sign changes (SSC), waveform length (WL), Willison amplitude (WAMP), root-mean-square (RMS), variance (VAR), v-order (V-ord, order of 3), log-detector (LogDet), autoregressive (AR1–AR6) coefficients, mean frequency (MnF), median frequency (MdF), peak frequency (PF), mean power (MP), and power spectrum descriptors (PSD1–PSD6). Error bars represent SE. (A) Extrinsic muscles, (B) intrinsic muscles, and (C) extrinsic and intrinsic muscles. [file Image_1.tif]
